# Letter to the editor: Added value of backward contact tracing for COVID-19

**DOI:** 10.2807/1560-7917.ES.2024.29.4.2400003

**Published:** 2024-01-25

**Authors:** Graham Fraser, Gareth Hughes, Simon Packer, Obaghe Edeghere, Isabel Oliver

**Affiliations:** 1 United Kingdom Health Security Agency, London, United Kingdom

**Keywords:** COVID-19, contact tracing, backward contact tracing, pilot study

**To the editor:** Boelsums et al. report [1] that their pilot study on the addition of manual backward contact tracing (BCT) did not provide any added value to conventional tracing for COVID-19 in the Rotterdam-Rijnmond area of the Netherlands. We think there may be explanations for this finding other than their conclusion that manual BCT is inherently ineffective.

In their study, the BCT evaluation took place during a period of almost total lockdown (only schools were open). Over half of all persons identified by cases as possible sources of their infection were already registered as confirmed cases in the Dutch Public Health Services database. Most were contacts from household, school or work settings where, as the authors observe, individuals tend to self-test when a new case occurs. From first principles, modelling and empirical evidence [2,3], BCT is expected to be most effective for detecting chains of infection otherwise unknown to public health authorities, particularly in community settings, e.g. in the hospitality and leisure sector.

The prevalent variant of the severe acute respiratory syndrome coronavirus 2 (SARS-CoV-2) may also have been a factor. Transmission of the Alpha variant (and subsequent variants) have shown less overdispersion than the original virus [4]. Backward contact tracing is expected to be less effective with pathogens which are not over-dispersed (backward traced contacts identified are less likely to have infected multiple individuals), and where a higher proportion of transmission events are likely to be due to household exposure.

Some decisions on the intervention design may have been influential. The decision not to test asymptomatic potential source contacts could have been influential, given that approximately one third of all COVID-19 transmission is thought to be from asymptomatic cases [5], and the prevalence among contacts identified as the possible source of someone’s infection is likely to be even higher. The authors of the only reported analytical epidemiological study of BCT found that positivity rates for backward traced contacts were similar to those identified through conventional tracing, and argued that it was cost effective to test and quarantine all backward traced contacts found [6]. The exclusion of suspect locations from further investigation may also have been a missed opportunity; numerous cluster investigations report this empirical approach, and some public health authorities have recommended such targeted use of BCT. Backward contact tracing was successfully implemented as part of the national surveillance and tracing programme in Japan during the first year of the pandemic; several Asian countries in particular reported the targeted use of BCT in investigations of case clusters [3,7,8].

Empirical as well as theoretical evidence to date show that BCT is likely to be most effective for respiratory pathogens characterised by over-dispersed transmission (particularly for initial pandemic variants), deployed when incidence is low (before and after pandemic waves), and during periods other than lockdown when community as well as institutional and domestic exposures are important. Backward contact tracing interventions which investigate suspect locations as well as named individuals are likely to be most effective.

In focusing on evaluation of the manual phases of BCT, the authors passed over the substantial informatics achievement of the programme. Inclusion of a ‘source question’ in contact tracing is the foundation of any BCT programme or intervention. An intrinsic value of BCT is its contribution to understanding transmission: when and how it is occurring as well as its operational utility. Its deployment here – at a time when the World Health Organization (WHO) recommended forward contact tracing only – would undoubtedly have provided Dutch authorities with invaluable intelligence for pandemic management and policy, which would have been even greater outside lockdown periods.

We agree with the authors’ salutary reminder that theoretical models do not necessarily equivalently transfer to real-world interventions, which require careful attention to context – including timing and variant characteristics – and design.
